# Amelioration of Central Nervous System Autoimmunity Through FFAR2 Agonism Is Associated With Changes in Gut Microbiota

**DOI:** 10.1002/brb3.70350

**Published:** 2025-02-28

**Authors:** Bojan Jevtić, Goran Stegnjaić, Suzana Stanisavljević, Milica Lazarević, Filip Nikolić, Graeme L. Fraser, Đorđe Miljković, Mirjana Dimitrijević

**Affiliations:** ^1^ Department of Immunology, Institute for Biological Research “Siniša Stanković”–National Institute of Republic of Serbia University of Belgrade Belgrade Serbia; ^2^ Department of Plant Physiology, Institute for Biological Research “Siniša Stanković”–National Institute of Republic of Serbia University of Belgrade Belgrade Serbia; ^3^ Epics Therapeutics S.A. Gosselies Belgium

**Keywords:** experimental autoimmune encephalomyelitis, free fatty acid receptor 2, gut microbiota, short‐chain fatty acids

## Abstract

**Purpose**: The intestinal immune compartment is increasingly recognized as an important player in central nervous system (CNS) autoimmunity. We have recently reported that oral administration of the free fatty acid receptor 2 (FFAR2) agonist Cpd1 in the inductive phase of experimental autoimmune encephalomyelitis (EAE) in rats ameliorates the disease by stimulating the regulatory immune response in the intestine.

**Method**: Here, the effects of Cpd1 on the gut microbiota and short‐chain fatty acid (SCFA) composition were investigated in the same experimental system.

**Finding**: Increased levels of the phylum *Proteobacteria*, the class *Gammaproteobacteria*, the orders *Burkholderiales* and *Erysipelotrichales*, the families *Sutterellaceae* and *Erysipelotrichaceae*, and the genera *Parasutterella* and *Faecalibaculum* were observed in agonist‐treated rats. The genera *Allobaculum* and *Ileibacterium* were only detected in the agonist‐treated group. The treatment led to changes in the functional profile of the gut microbiota both in the KEGG orthologous pathways and in the clusters of orthologous genes. In addition, an altered profile of intestinal SCFA content was observed in the agonist‐treated group.

**Conclusion**: The effects of Cpd1 on the gut microbiota and SCFA composition are relevant to the observed treatment benefit of FFAR2 agonism in the EAE model during the inductive phase of the disease.

## Graphical abstract

DA rats immunized with spinal cord homogenate (SCH) develop experimental autoimmune encephalomyelitis (EAE) which is ameliorated by the treatment of rats with the FFAR2 agonist Cpd1 in the inductive phase of the disease. Changes in gut microbiota (16S) and short chain fatty acids (SCFA) related to the disease amelioration are determined in DA rats treated with Cpd1.

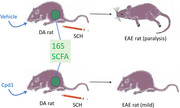

## 1 Introduction

Free fatty acid receptor 2 (FFAR2), also known as GPR43, is a G‐protein–coupled receptor that recognizes short‐chain fatty acids, such as acetate, propionate, and butyrate. It is expressed on immune cells of the intestine, including the major controllers of the immune response, regulatory T cells (Treg) (Smith et al. 2013). The dominant expressers of FFAR2 in the gut innate immune compartment are innate lymphoid cells (ILC), ILC3 in particular (Chun et al. 2019). ILC3 have been established as potent immunoregulatory cells (Chun et al. 2019; Zhou et al. 2019), and their role in preventing and modulating central nervous system (CNS) autoimmunity was proposed (Miljković et al. 2021). Importantly, cooperation between ILC3 and Treg is essential for maintaining immune homeostasis in the intestine (Zhou et al. 2019; Lyu et al. 2022), whereas a general disbalance of the gut immune compartment is strongly linked with autoimmunity (Q. Wang et al. 2023). Both Treg and ILC3 respond to FFAR2 activation by expanding cell population and functional activity (Smith et al. 2013; Chun et al. 2019). Thus, FFAR2 stimulation in intestinal immune cells is a plausible way to counteract autoimmunity. Indeed, we have previously shown that treatment of Dark Agouti (DA) rats in the inductive phase of experimental autoimmune encephalomyelitis (EAE) with an FFAR2 agonist (Cpd1) leads to a reduction in the CNS‐directed autoimmune response and corresponding treatment benefit in this disease model (Lazarević et al. 2024).

Gut microbiota dysbiosis has been implicated in the pathogenesis of multiple sclerosis and EAE (Sivaprakasam et al. 2016; Schumacher et al. 2024). Notably, FFAR2 signaling has been identified to be of importance for promoting gut microbiota homeostasis (Turner et al. 2024). Also, the beneficial effects of numerous treatments acting through modulation of CNS autoimmunity are additionally associated with the changes in the intestinal microbiota (Khadka et al. 2021; Sell et al. 2022; Han et al. 2024). SCFAs are among the major metabolic products of gut microbiota, and as such, they have tremendous effects on the intestinal immune compartment (Martin‐Gallausiaux et al. 2021; Li et al. 2018). Importantly, SCFAs stimulate the regulatory immune response and are proposed to counteract development of CNS autoimmunity as a component of the observed therapeutic effect (Luu et al. 2020; Schiweck et al. 2022).

Previously, we have demonstrated the efficacy of the FFAR2 agonist (Cpd1) in amelioration of EAE (Lazarević et al. 2024) and the importance of the gut microbiota in CNS autoimmunity in rats (Stanisavljević, Lukić, Momčilović, et al. 2016; Stanisavljević, Lukić, Soković, et al. 2016). The goal of the current study is to determine the effect of Cpd1 on the composition of gut microbiota and SCFA in DA rat EAE. These study endpoints are relevant to preservation of gut immune homeostasis as a contributing factor in the treatment of autoimmune diseases.

## 2 Methods

### 2.1 Animals, EAE Induction and Treatment

DA rats were bred and maintained in the animal facility of the Institute for Biological Research “Siniša Stanković.” The experiments were performed with permission granted by the Veterinary Administration of the Ministry of Agriculture, Forestry and Water Management of the Republic of Serbia (Nos. 323–07‐01337/2020–05 and 323–07‐05815/2020–05/1). Nineteen 6‐month‐old female DA rats were immunized with spinal cord homogenate (SCH) without complete Freund's adjuvant (CFA). FFAR2 agonist (“Cpd1,” compound 1 in patent no. WO 2011/073376 A1, provided by Epics Therapeutics S.A. under Material Transfer Agreement permitting use of the compound) (Forbes et al. 2015; Hoveyda et al. 2011) was used for the treatment, as previously described (Lazarević et al. 2024). Two groups of rats received 15 mg/kg of Cpd1 (EAE + Cpd1) daily or distilled water as vehicle (EAE) by oral gavage starting on the day of immunization and finishing on Day 5 after immunization.

### 2.2 Microbial DNA Extraction and 16S rRNA Sequencing

The animals were sacrificed on Day 5 after immunization, and the terminal ileum was isolated by excising the distal end of the small intestine, approximately 2–3 cm long. Ileal contents were collected, and genomic DNA (gDNA) was extracted using the ZymoBIOMICS DNA Miniprep Kit Cat. D4304 (Zymo Research, Irvine, CA, USA) following the manufacturer's instructions. Quantity and purity of the isolated DNA were measured with NanoPhotometer N60 (Implen, Munich, Germany). All samples were diluted to a concentration of 10 ng/mL in a 60 µL total volume and sent to Novogene Co. (Cambridge, UK) for full‐length 16S amplicon metagenomics library preparation. The library was checked with Qubit for quantification. Quantified libraries were pooled and sequenced on PacBio Sequel II/IIe systems (PacBio, Menlo Park, CA). Bioinformatic analysis was performed by Novogene Co. Lima software was used to differentiate samples by barcode sequences, and CCS (SMRT Link v7.0) corrected the sequences. Sequences shorter than 1340 bp or longer than 1640 bp were removed. SSR filtration and primer removal were done using cutadapt, resulting in clean reads for amplicon sequence variants (ASVs) analysis and species annotation in QIIME2 software (Version QIIME2‐202006). Phylogenetic relationships and dominant species differences were analyzed with multiple sequence alignment in QIIME2. ASVs abundance was normalized for α‐ and β‐diversity analyses. α‐Diversity (a measure of microbiota diversity) was calculated using five indices: Observed_otus, Chao1, Shannon, Simpson, and Dominance (Willis 2019). β‐Diversity (a measure of the similarity or dissimilarity of two communities) was calculated based on weighted and unweighted UniFrac distances in QIIME2 (Lozupone et al. 2007). Principal coordinate analysis (PCoA) was used to visualize sample differences by transforming UniFrac distances into orthogonal axes, with the results displayed in QIIME2 and R software (Version 2.15.3). A Venn diagram was created in QIIME2 to illustrate unique and shared ASVs. Community structure differences between groups were analyzed using adonis and anosim functions in QIIME2, while MetaStat and *t*‐test analysis in the R software (Version 3.5.3) were used to find out the significantly different species at each taxonomic level. LEfSe (Version 1.0) identified biomarkers with an LDA score threshold of 4. Finally, functional annotation of the communities was performed using PICRUSt2 (Version 2.1.2‐b).

### 2.3 Quantification of SCFA

SCFAs were isolated from cecal content on Day 5 after immunization, according to a modified version of a published protocol (S. Zhang et al. 2019). In brief, animals were euthanized, and the cecum was isolated and stored at −20°C until SCFA extraction. On the day of extraction, 100 mg of the cecal contents were weighed and mixed with 1 mL of deionized water in a 2‐mL tube. Samples were vortexed for 1 min and then sonicated in an ice‐water ultrasonic bath at 40 kHz for 10 min, followed by centrifugation at 10,000 × *g* for 15 min. Subsequently, 500 µL of the supernatant was transferred to a new 2‐mL tube, mixed with 50 µL of 5 M HCl, and vortexed. The mixture was extracted with 500 µL anhydrous diethyl ether (DE) (1:1, v/v), vortexed, incubated on ice for 5 min, and centrifuged at 10,000 × *g* for 5 min at 4°C. The DE layer (upper phase) was carefully transferred to a new 2‐mL tube containing anhydrous sodium sulfate (Na_2_SO_4_) to remove residual water. This extraction process was repeated three times to ensure efficient SCFA recovery. The DE layers were pooled and derivatized for further analysis.

Profiling of eight targeted SCFAs (acetic, propionic, butyric, valeric, 4‐methyl valeric, hexanoic, and heptanoic acids) was performed using an Agilent 8890 gas chromatography (GC) system coupled with a Mass Selective Detector (5977B GC/MSD, Agilent Technologies, Santa Clara, CA) and connected to an automated sample extraction and enrichment platform (Centri, Markes International Ltd., Bridgend, UK). The derivatization procedure was carried out by transferring 100 µL of DE extract to a GC vial and then adding 1 µL of BSTFA reagent and shaking. The obtained mixture was kept in a GC vial for 15 min at room temperature. Then, the derivatized samples were applied to the GC column. The same derivatization procedure was carried out for the standards. Chromatographic separations were performed for 21 min on an HP‐5MS column (30 m × 0.25 mm, 0.25 µm film thickness; Agilent Technologies). Helium (99.999%, The Linde Group, Ireland) was used as a carrier gas at a flow rate of 1.6 mL/min. The transfer line was heated at 280°C, and the detector temperature was set to 270°C. Mass spectra were acquired in the positive EI mode (+70 eV), with the temperature of the EI source set to 280°C. Column temperature was linearly programmed from 40°C to 300°C at a rate of 20 mL/min and held isothermally at 240°C for the subsequent 5 min. Analyses were performed in a combined single ion monitoring (SIM)/SCAN mode. SCAN mode enabled the tracking of compounds within the range of 45–500 amu and their identification by comparing their mass spectra and retention times with those of the respective standards, as well as with spectra within the NIST05 library. Quantification of compounds of interest was based on the SIM data targeted toward the following masses: 60.06, 74.08, 88.11, 102.13, 116.16, and 130.18. The external standard method was adopted using the calibration curves of the pure standards, which all revealed good linearity with *r* ^2^ values exceeding 0.99 (peak areas vs. concentration). Standards of acetic, propionic, butyric, valeric, 4‐methyl valeric, hexanoic, and heptanoic acids were commercially purchased from Sigma‐Aldrich (St. Louis, MO).

### 2.4 Statistical Analysis

GraphPad Prism 9 software (GraphPad Software, San Diego, California, USA) was used for statistical analysis. The data were presented in histograms (mean + SD), box and whisker diagrams (minimum to maximum), and pie charts (mean). The significance of differences between groups was determined using a two‐tailed Student's *t*‐test, as indicated in the figure legends. *p* ≤ 0.05 was considered statistically significant.

## 3 Results

### 3.1 The Effect of Cpd1 Treatment on the Gut Microbiome Diversity in EAE Rats

**FIGURE 1:**
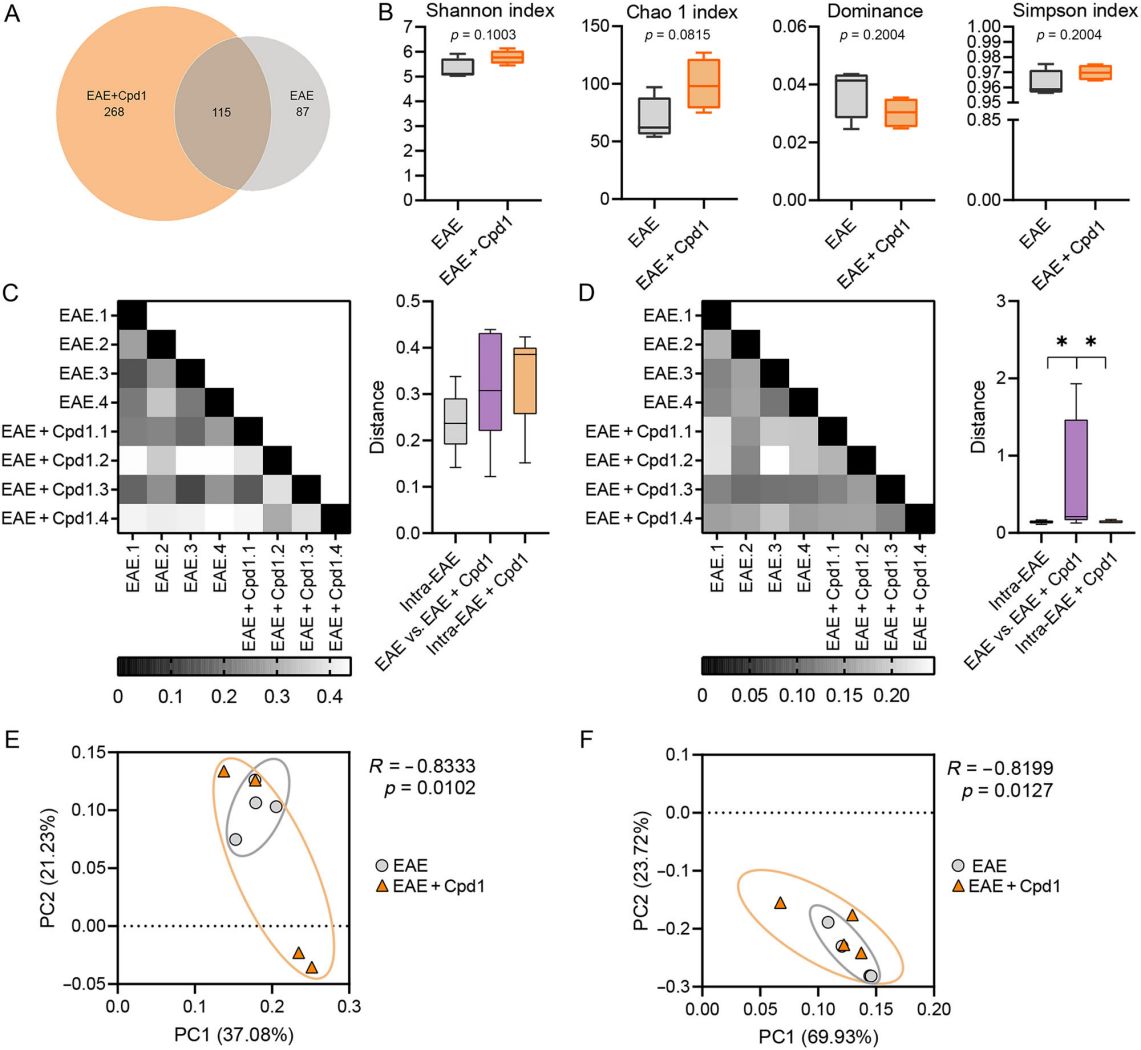
Diversity of the gut microbiota in Cpd1‐ versus vehicle‐treated rats. (A) Venn diagram showing the number of exclusive and shared amplicon sequence variants (ASVs) in the gut microbiota of Cpd1 + EAE and EAE groups of rats. (B) Alpha diversity indices: Shannon index, Chao1 index, Dominance, and Simpson index. Heatmaps and boxplots of sample distance based on (C) unweighted and (D) weighted UniFrac analysis at the ASV level. (E) Unweighted and (F) weighted principal coordinate analysis (PCoA) plots. The percentage of variance explained by each principal coordinate is shown for PC1 and PC2 (*N* = 4, *p < 0.05; unpaired *t*‐test with Welch's correction).

Analysis of 16S rRNA sequencing revealed different microbial populations in EAE and Cpd1‐treated EAE rats. The Venn diagram in the ASV levels depicted 115 common species and 288 and 87 species specific to the Cpd1‐treated EAE and EAE groups, respectively (Figure 1A). However, there were no significant differences between the groups in terms of α‐diversity metrics, including Shannon, Chao1, Dominance, and Simpson indices (Figure 1B). UniFrac distance analysis and PCoA of 16S data were used to assess β‐diversity in the gut microbiome of EAE and Cpd1‐treated EAE rats. There were no differences between pairwise comparisons of unweighted UniFrac distances based on the presence/absence of different taxa within each group and between groups (Figure 1C). However, the weighted UniFrac distance based on the presence/absence of different taxa and their abundance was significantly higher between groups than the distances within each group (Figure 1D). To assess similarity between groups, PCoA plots were generated from the unweighted and weighted UniFrac distance metrics. The analysis of similarities (ANOSIM) showed significant similarities between the composition of the group communities with negative *R* values (Figure 1E,F), indicating the lack of variability between the groups.

### 3.2 The Effect of Cpd1 Treatment on the Composition of the Gut Microbiome in EAE Rats

**FIGURE 2:**
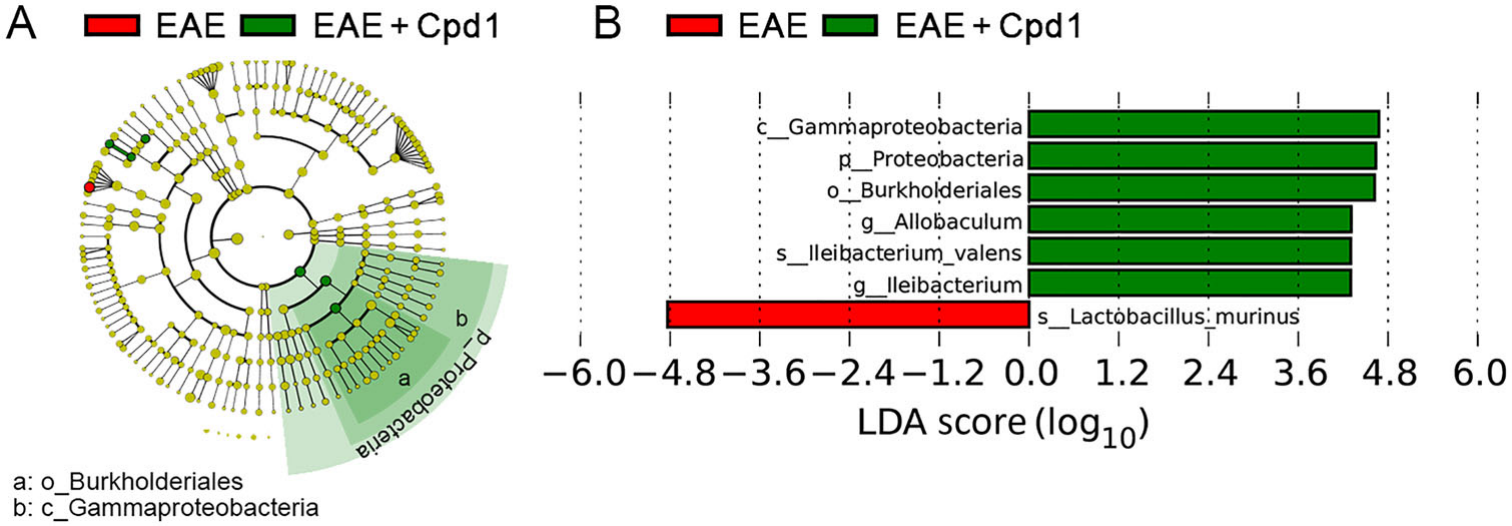
Linear discriminant analysis effect size (LEfSe) analysis to identify differences in abundant taxa in the feces of Cpd1‐ versus vehicle‐treated rats. (A) The cladogram shows the phylogenetic relationship of the significant amplicon sequence variants associated with each group. The yellow circles in the cladogram represent bacterial taxa that did not differ significantly. (B) Taxonomic groups with LDA scores > 4.0 with *p* ≤ 0.05. Red and green indicate taxa enriched in vehicle versus Cpd1 groups, respectively. c, class; f, family; g, genus; o, order; p, phylum.

In addition, we investigated the overall microbiota community differences from phylum to species levels in EAE rats treated with Cpd1 versus vehicle controls with the aim of identifying potential biomarkers in the gut microbiome responsive to Cpd1 treatment. The phylogenetic relationship of significant ASVs associated with each group is shown in Figure 2A. LefSe analysis identified the predominant bacterial biomarkers contributing to the group differences. Specifically, the Cpd1‐treated group exhibited a significant increase (LDA > 4) in the relative abundance of *Proteobacteria* (at the phylum level), *Gammaproteobacteria* (at the class level), *Burkholderiales* (at the order level), *Allobaculum* and *Ileibacterium* (at the genus level), and *Ileibacterium*_*valens* (at the species level) (Figure 2B). On the other hand, a significant decrease of *Lactobacillus*_*murinus* (at species level) was observed in Cpd1‐treated rats versus vehicle controls (Figure 2B).

**FIGURE 3:**
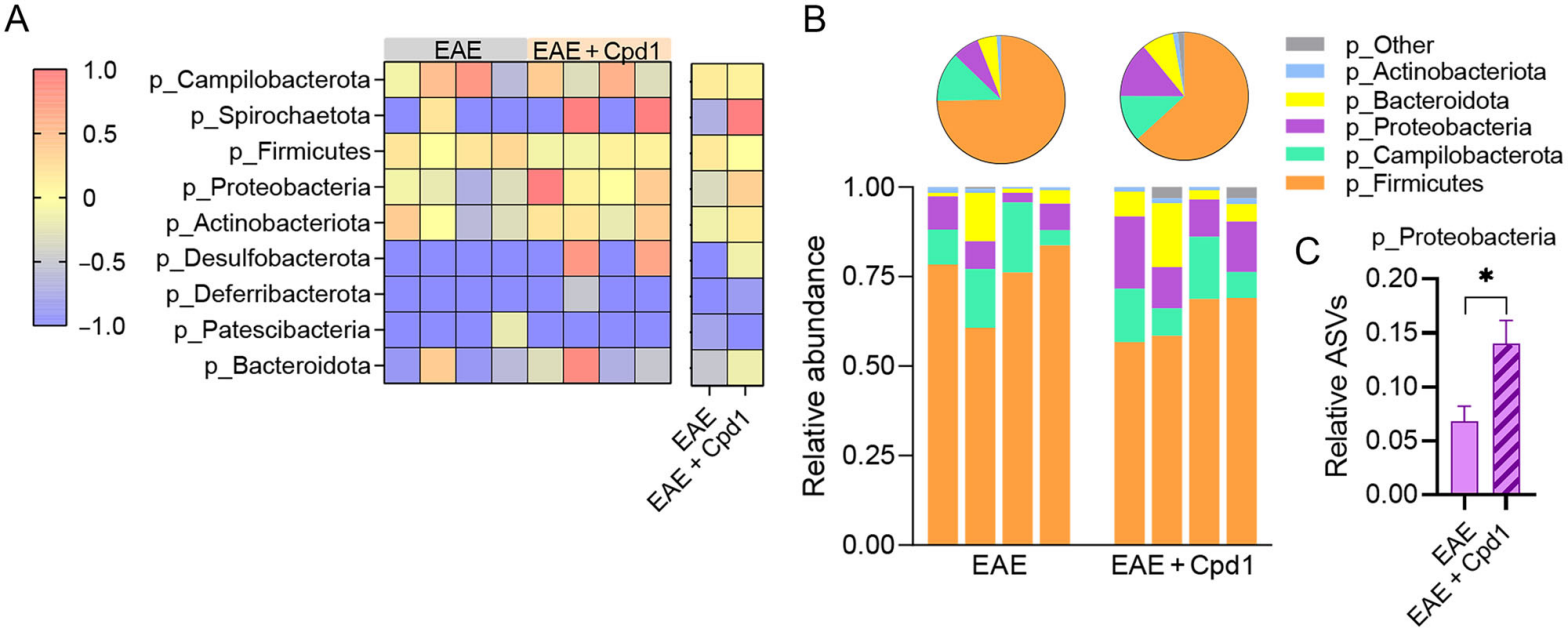
Diversity of the gut microbiota at the phylum level in Cpd1‐ versus vehicle‐treated rats. (A) Heatmap showing the composition of amplicon sequence variants (ASVs) identified in each sample. (B) Stacked bars and pie charts show the relative abundance of ASVs with a mean > 0.01. (C) Histogram shows the relative expression (mean + SD) of p_Proteobacteria (*N* = 4, *p ≤ 0.05, *t*‐test).

Phylum‐level taxonomic classification revealed that nine phyla were represented in the EAE and Cpd1‐treated EAE groups (Figure 3A), with most ASVs belonging to the phylum *Firmicutes*, followed by the phyla *Campilobacterota* and *Proteobacteria* (Figure 3B). The relative amounts of specific phyla were similar in both groups, except for the amount of ASVs representing the phylum *Proteobacteria*, which increased significantly by Cpd1 treatment in the EAE group (Figure 3C).

**FIGURE 4:**
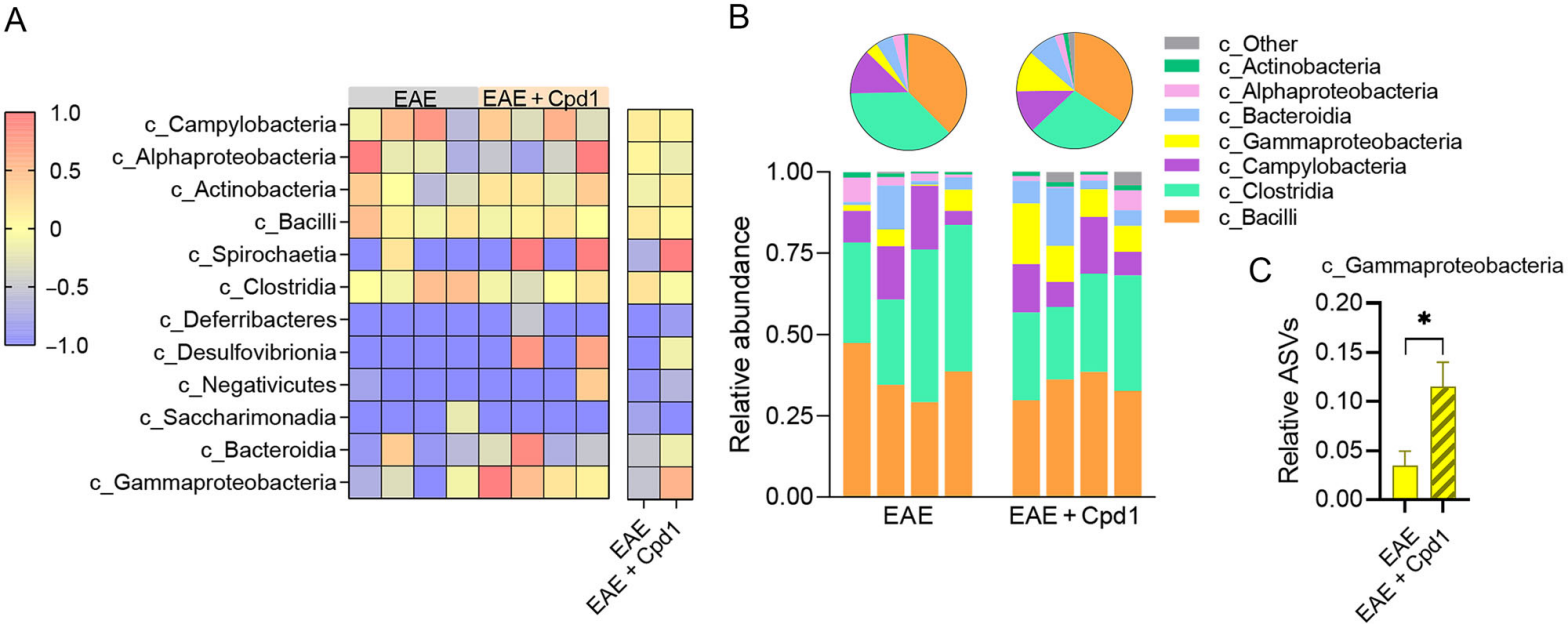
Diversity of the gut microbiota at the class level in Cpd1‐ versus vehicle‐treated rats. (A) Heatmap showing the composition of amplicon sequence variants (ASVs) identified in each sample. (B) Stacked bars and pie charts show the relative abundance of ASVs with a mean > 0.01. (C) Histogram shows the relative expression (mean + SD) of c_Gammaproteobacteria (*N* = 4, *p < 0.05, *t*‐test).

Among the 12 taxonomic classes identified (Figure 4A), the *Bacilli*, *Clostridia*, and *Campylobacteria* classes dominated in EAE rats regardless of Cpd1 treatment (Figure 4B). However, a higher relative expression of ASVs representing *Gammaproteobacteria* was detected in Cpd1‐treated EAE rats compared to EAE rats (Figure 4C).

**FIGURE 5:**
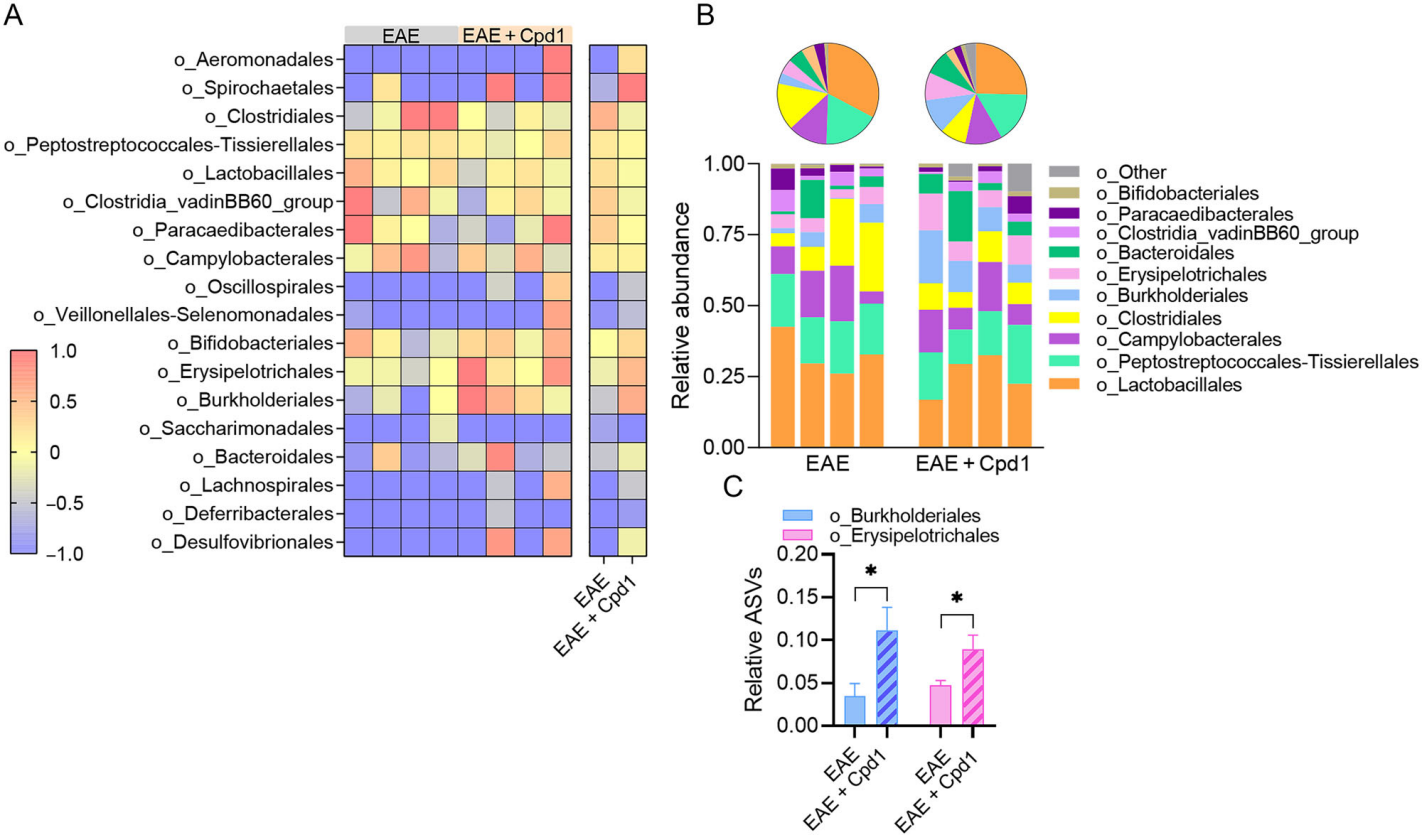
Diversity of the gut microbiota at the order level in Cpd1‐ versus vehicle‐treated rats. (A) Heatmap showing the composition of amplicon sequence variants (ASVs) identified in each sample. (B) Stacked bars and pie charts show the relative abundance of ASVs with a mean > 0.01. (C) Histograms show the relative expression (mean + SD) of o_Burkholderiales and o_Erysipelotrichales (*N* = 4, *p < 0.05, *t*‐test).

Taxonomic classification at the order level revealed 18 orders (Figure 5A), with six orders, that is, *Lactobacillales*, *Peptostreptococcales‐Tissierallales*, *Campylobacterales*, *Clostridiales*, *Burkholderiales*, and *Erysipelotrichales*, accounting for more than three‐fourths of all ASVs identified in Cpd1‐treated EAE rats and EAE rats (Figure 5B). Cpd1 treatment in EAE rats increased the relative expression of ASVs related to *Burkholderiales* and *Erysipelotrichales* compared to EAE rats (Figure 5C).

**FIGURE 6:**
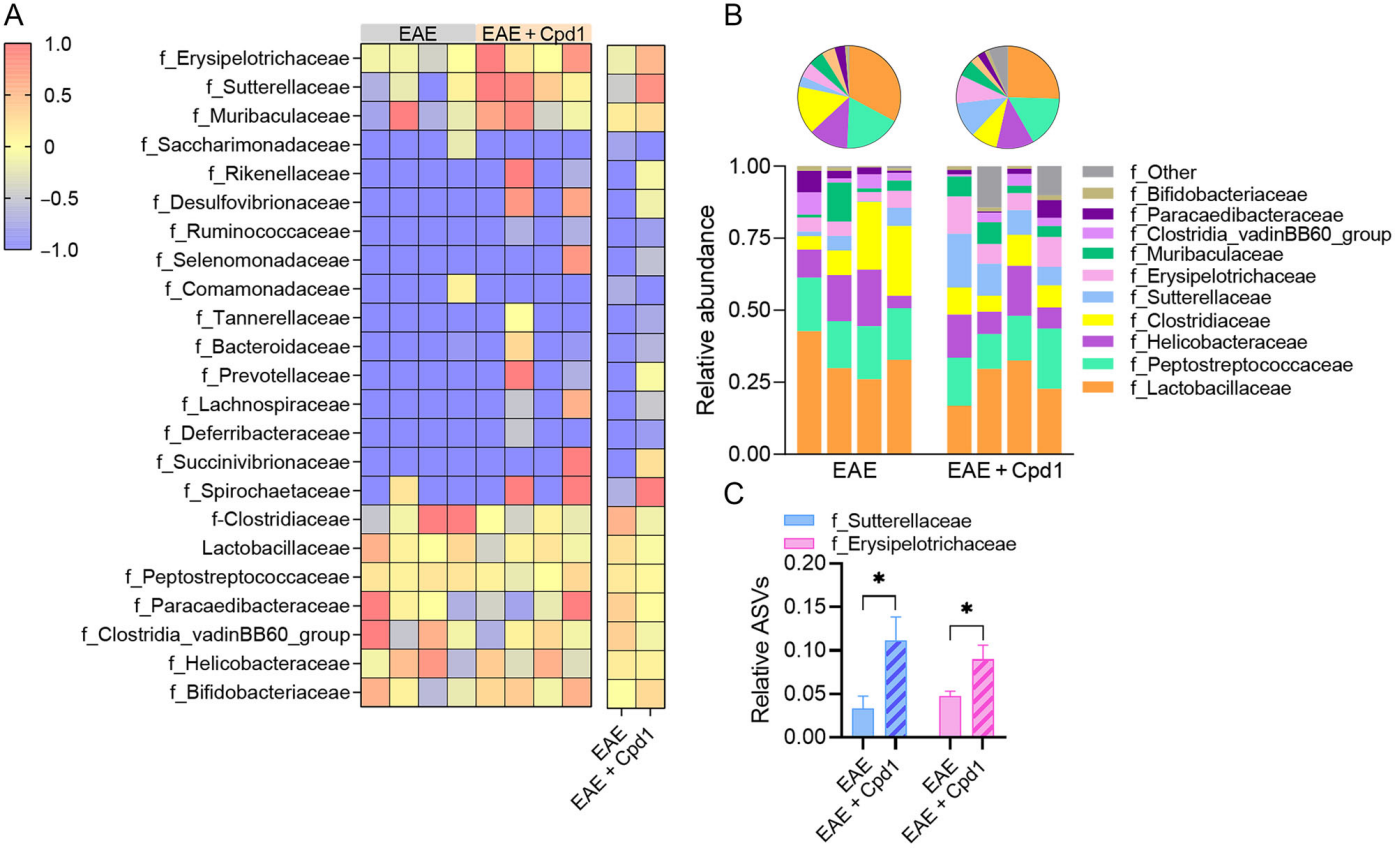
Diversity of the gut microbiota at the family level in Cpd1‐ versus vehicle‐treated rats. (A) Heatmap showing the composition of amplicon sequence variants (ASVs) identified in each sample. (B) Stacked bars and pie charts show the relative abundance of ASVs with a mean > 0.01. (C) Histograms show the relative expression (mean + SD) of f_Sutterellaceae and f_Erysipelotrichaceae (*N* = 4, *p < 0.05, *t*‐test).

Taxonomic analysis at the family level revealed 23 families (Figure 6A), including six dominant families, that is, *Lactobacillaceae*, *Peptostreptococcaceae*, *Helicobacteraceae*, *Clostridiaceae*, *Sutterellaceae*, and *Erysipelotrichaceae*, which accounted for more than three‐fourths of the ASVs in both Cpd1‐treated EAE rats and EAE rats (Figure 6B). Treatment with Cpd1 in EAE rats increased the relative expression of ASVs related to *Sutterellaceae* and *Erysipelotrichaceae* compared to EAE rats (Figure 6C).

**FIGURE 7:**
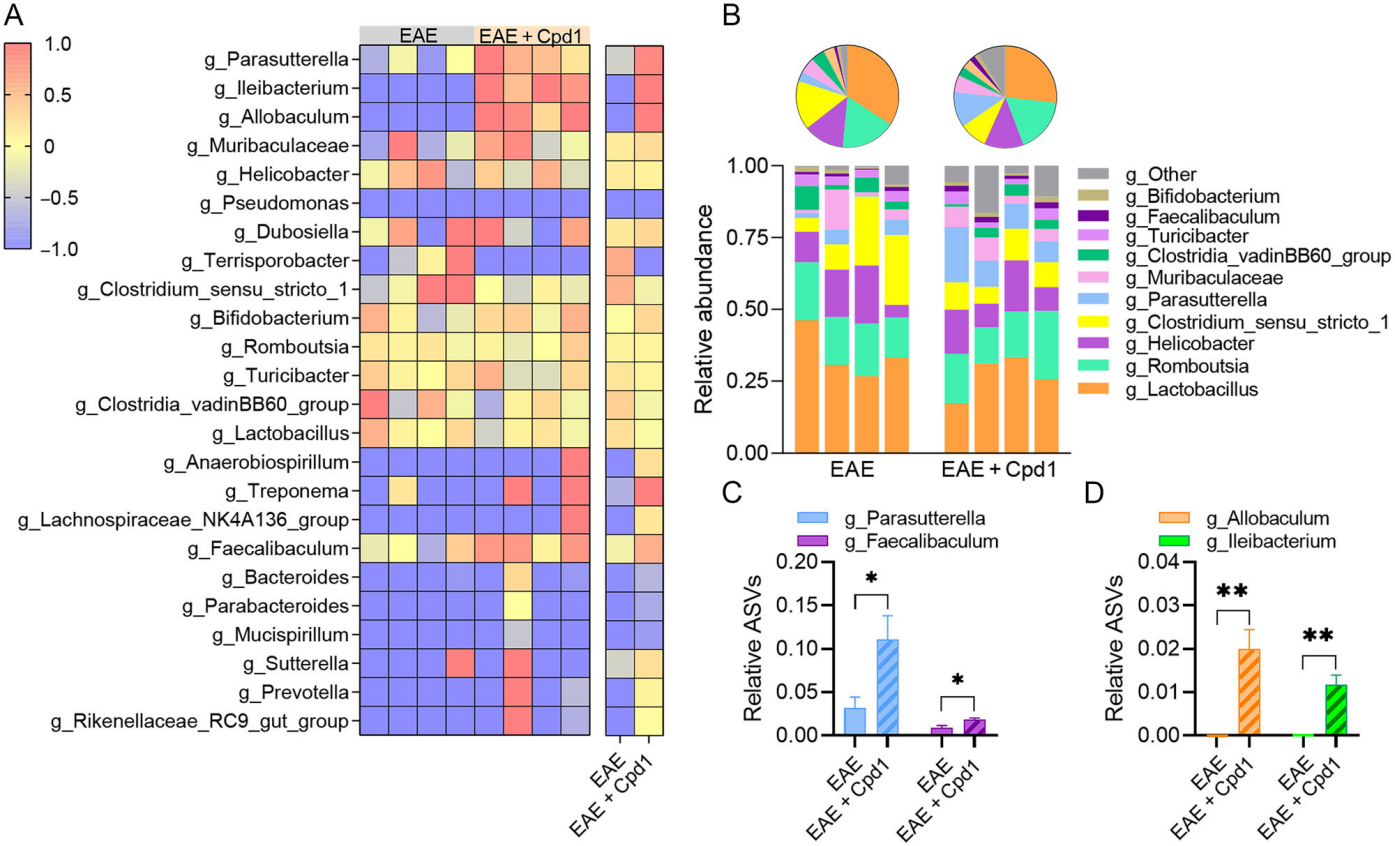
Diversity of the gut microbiota at the genus level in Cpd1‐ versus vehicle‐treated rats. (A) Heatmap showing the composition of amplicon sequence variants (ASVs) identified in each sample. (B) Stacked bars and pie charts show the relative abundance of ASVs with a mean > 0.01. (C) Histograms show the relative expression (mean + SD) of g_Parasutterella and g_Faecalibaculum (*N* = 4, *p ≤ 0.05, *t*‐test). (D) Histograms show the relative expression (mean + SD) of g_Allobaculum and g_Ileibacterium (*N* = 4, **p < 0.01, *t*‐test).

The gut microbiota of both Cpd1‐treated EAE rats and EAE rats was characterized by the expression of 24 different taxa at the genus level (Figure 7A), with the most abundant ASVs belonging to the genera *Lactobacillus*, *Romboutsia*, *Helicobacter*, *Clostridium_sensu_stricto_1*, and *Parasutterella* (Figure 7B). Among the dominant genera, a significant increase in *Parasutterella* genera was observed in Cpd1‐treated rats compared to EAE rats (Figure 7C). Higher expression of ASVs was also detected for the genus *Faecalibaculum* (Figure 7C). In addition, two genus taxa, *Allobaculum* and *Ileibacterium*, were only detected in Cpd1‐treated EAE rats but not in EAE rats (Figure 7D).

### 3.3 The Effect of Cpd1 Treatment on the Functional Profile of the Gut Microbiota in EAE Rats

**FIGURE 8:**
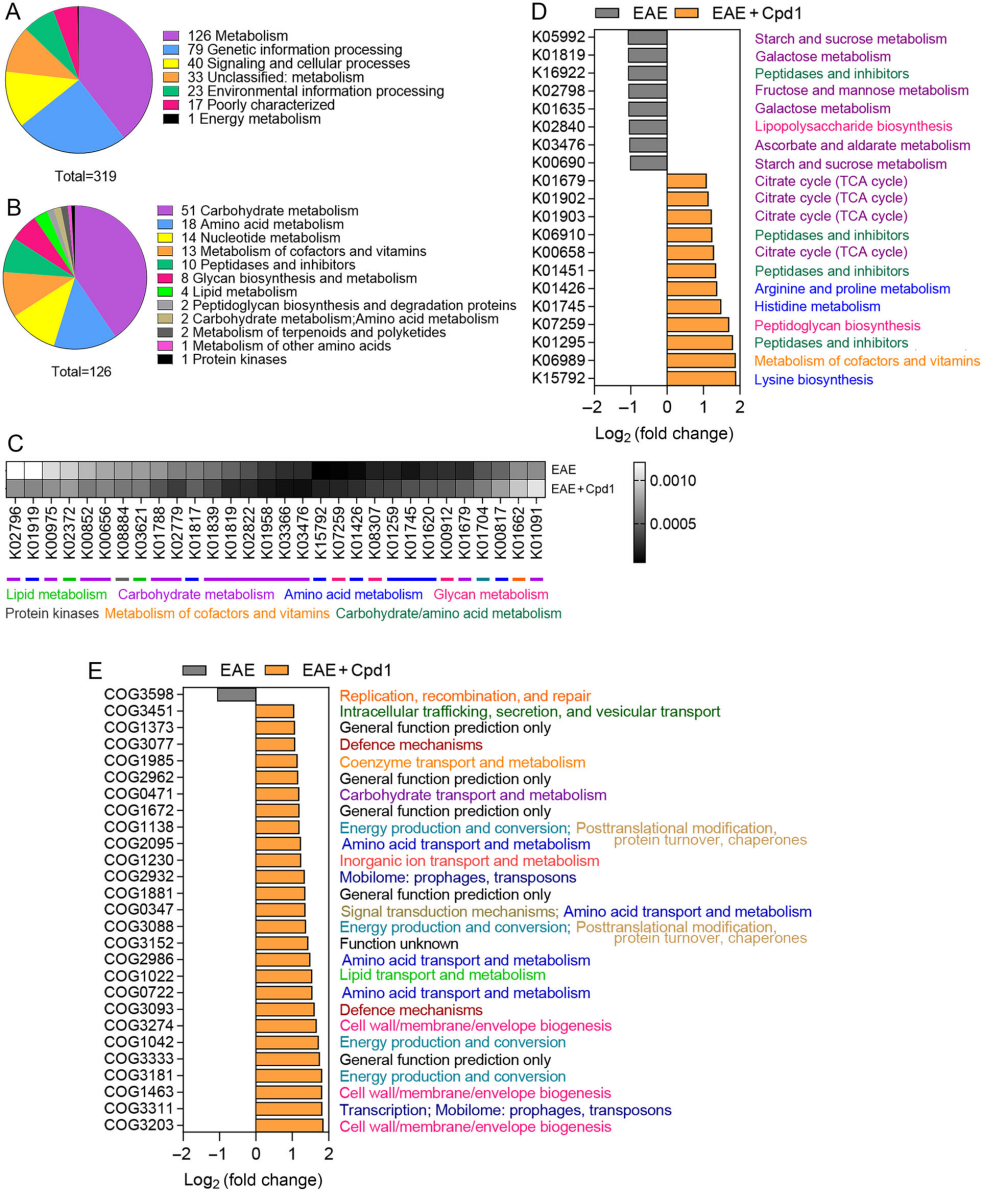
Functional analysis of the gut microbiome in Cpd1‐ versus vehicle‐treated rats. Distribution of KEGG Orthology (KO) (A) pathways (*n* = 319) and (B) metabolism‐related pathways (*n* = 126) differentially expressed in Cpd1‐treated EAE rats and EAE rats. (C) Heatmap of differential expression of KO pathways related to metabolism (top 30) between Cpd1‐treated EAE rats and EAE rats. *p* ≤ 0.05. (D) Upregulated and downregulated metabolism‐related KO pathways ranked by fold changes in relative abundance (|log_2_ (fold change)| ≥ 1), *p* ≤ 0.05. (E) Upregulated and downregulated COG pathways ranked by fold changes in relative abundance (|log_2_ (fold change)| ≥ 1), *p* < 0.05.

PICRUSt was used to assess the functional content of the gut microbiota based on the 16S data. Functional analyses of the gut microbiome of Cpd1‐treated EAE rats and EAE rats showed differential expression of 319 of 1702 identified KEGG orthology (KO) pathways (*p* ≤ 0.05) between groups (Figure 8A; Supporting Information S1). The differential expression included 126 KO metabolism‐related pathways grouped into 12 categories (Figure 8B). Amino acid metabolic pathways, glycan metabolic pathways, and metabolism of cofactors and vitamins were enriched, whereas carbohydrate metabolic pathways and lipid metabolic pathways were reduced in the Cpd1‐treated EAE group (Figure 8C). Figure 8D shows the most significant differences in the abundance of KO metabolic pathways in the Cpd1–EAE group compared with the EAE group. As for the richness of COG categories, Cpd1 treatment increased a total of 26 COG categories, including amino acid transport and metabolism, energy production and conversion, cell/wall/membrane biogenesis, and defense mechanism (Figure 8E).

### 3.4 The Effect of Cpd1 Treatment on SCFA Production in the Gut of EAE Rats

**FIGURE 9:**
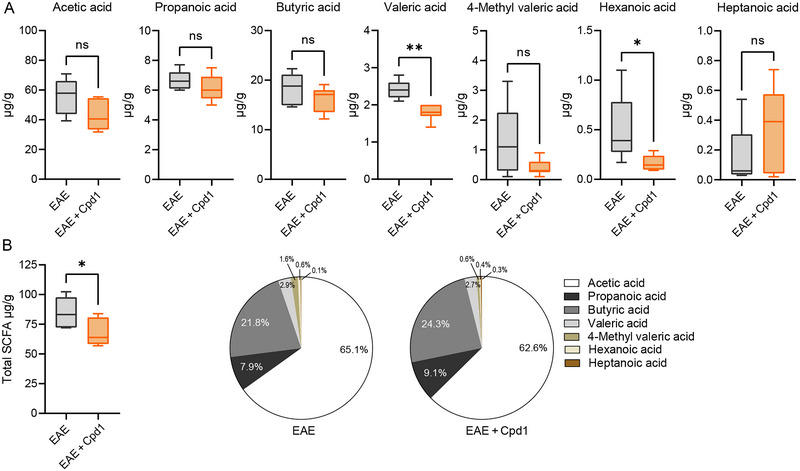
Production of fatty acids by the gut microbiome in Cpd1‐ versus vehicle‐treated rats. (A) Concentrations of acetic acid, propanoic acid, butyric acid, valeric acid, 4‐methyl valeric acid, hexanoic acid, and heptanoic acid in the cecal contents of Cpd1‐treated EAE rats and EAE rats. (B) Total and relative short‐chain fatty acid (SCFA) concentration in the cecal contents of Cpd1‐treated EAE (*N* = 6) and EAE (*N* = 5) rats (*p < 0.05, **p < 0.01, *t*‐test).

The SCFA content was measured in the content of the cecum. The concentrations of valeric acid and hexanoic acid were lower in the EAE group treated with Cpd1 compared to the EAE group (Figure 9A). The total SCFA content decreased, but the relative amount of propanoic acid increased (*p* = 0.02) in the EAE group treated with Cpd1 compared to the EAE group (Figure 9B). Although statistical significance was detected, the changes were minor.

## 4 Discussion

Our results extend knowledge of the mechanisms behind Cpd1‐imposed EAE amelioration. Specifically, we present that Cpd1 treatment in the inductive phase of EAE leads to changes in gut microbiota composition and minor changes in the relative abundance of SCFA in intestinal content.

Cpd1 treatment of EAE rats did not change the bacterial communities in the terminal ileum in terms of α‐ and β‐diversity, but it significantly altered the composition of the microbiota. The enriched bacterial communities at different taxonomic levels in the EAE rats treated with Cpd1 included *Proteobacteria* (phylum), *Gammaproteobacteria* (class), *Burkholderiales* (order), *Allobaculum* and *Ileibacterium* (genus), and *Ileibacterium*_*valens* (species). Changes in the composition of the gut microbiota preceded the development of EAE. Notably, Cpd1 treatment reduced mean, cumulative, and maximal score of EAE, as well as proportion of rats suffering from severe and lethal forms of the disease (Lazarević et al. 2024). Therefore, it can be assumed that the observed changes in the above mentioned bacterial taxa are associated with the protective effect of Cpd1 in EAE. While there is still no strict distinction between the beneficial and harmful intestinal microbiota in autoimmune and inflammatory diseases, there are reports corroborating our assumption. Recently, it has been reported that the enrichment of the *Proteobacteria* phylum in EAE mice under certain dietary regimes such as phytoestrogen‐free diet (Ghimire et al. 2022) and high‐fat diet (Shahi et al. 2022) has no beneficial effect on the disease. However, fecal microbiota transplantation reduced the severity of EAE in mice by increasing the relative abundance of *Proteobacteria* and *Firmicutes* and decreasing the abundance of *Bacteroides* and *Actinobacteria* phylum (S. Wang et al. 2021). In our study, Cpd1 treatment of EAE rats led to an increase in *Gammaproteobacteria* (class) and *Burkholderiales* (order), which have not been associated with autoimmune diseases in the past.

In addition, we showed that *Erysipelotrichales* (order) and *Erysipelotrichaceae* (family) were also enriched after treatment of EAE rats with Cpd1. This finding is consistent with a previously reported higher abundance of *Erysipelotrichaceae* in symptom‐free compared to severely diseased EAE mice (Moles et al. 2021). *Erysipelotrichaceae* species were reduced in MS patients compared to healthy controls (Cox et al. 2021; J. Chen et al. 2016). The potentially positive role of *Erysipelotrichaceae* CMM in MS was confirmed by the negative correlation with fatigue and the positive correlation with quality of life parameters (J. Chen et al. 2016). However, the above studies contradicted the increased susceptibility to induction of EAE in germ‐free mice monocolonized with *Erysipelotrichaceae* and attributed adjuvant‐like properties to these bacteria in the induction of Th17 cells (Miyauchi et al. 2020). The pathogenic role of Th17 cells from the gut‐associated lymphoid tissue in triggering EAE has been demonstrated, as their numbers increased significantly in EAE mice before the onset of clinical symptoms (Nouri et al. 2014). It is noteworthy that the *Erysipelotrichaceae* family belongs to the *Firmicutes* phylum, which was not altered by treatment with Cpd1 in EAE rats. The *Erysipelotrichaceae* family comprises numerous genera, including *Allobaculum* and *Faecalibaculum*, which were enriched in Cpd1‐treated EAE rats. Furthermore, the genus *Allobaculum* was undetectable in EAE rats. The increase in abundance of the genus *Allobaculum* was reported in norfloxacin‐induced suppression of EAE (H. Chen et al. 2019). In contrast, EAE mice have been shown to have low levels of the genus *Allobaculum* and microbial SCFA metabolites (Lin et al. 2024). Considering that *Allobaculum* and *Faecalibaculum* are important producers of butyrate (Zheng et al. 2021), one of the SCFAs with immunosuppressive function in the gut affecting various autoimmune diseases (Golpour et al. 2023), the amount of various SCFAs in cecal contents was measured. Although the total amount of SCFAs was reduced in EAE rats treated with Cpd1, the relative amount of butyrate slightly increased compared to that of EAE rats. In addition, the relative amount of propanoic acid was significantly higher in this group. Treatment with propanoic acid has been reported to improve clinical symptoms in mice suffering from EAE by increasing the number of Treg cells in the intestine and reducing the differentiation of Th17 cells (Haghikia et al. 2015). Functional analysis of the gut microbiota based on KO and COG metabolic pathways revealed no changes in SCFA metabolic pathways after treatment with Cpd1 in EAE rats. Therefore, it is unlikely that an altered microbiota contributes to Cpd1‐induced suppression of EAE through increased SCFA production, as the observed changes in SCFA levels are improbable to cause any important effect on FFAR2 signaling.

Indeed, we found decreased total SCFA levels in rats treated with Cpd1 but altered relative abundance of different SCFAs with significantly increased propanoic acid. It has been hypothesized that the genus *Allobaculum*, as an enormous glucose utilizer, reduces the availability of glucose to CD4^+^ T cells for proliferation and cytokine production in rheumatoid arthritis (Chimenti et al. 2015). Functional analyses of the gut microbiota in our study showed increased expression of several KO pathways, K01679, K01902, K01903, and K00658, belonging to the tricarboxylic acid (TCA) cycle in EAE rats treated with Cpd1. Thus, the increased carbohydrate and fatty acid degradation most likely caused a reduction in SCFA content in the cecum as well as increased energy consumption by the gut microbiota in Cpd1‐treated EAE rats compared to EAE rats. The functional analyses of the gut microbiota based on COG classification showed increased pathways for energy production and conversion, COG1138, COG3088, COG1042, and COG3181, in the same group. In addition, increased amino acid transport and metabolism, as well as increased arginine, proline, and histidine metabolism, were found in the COG and KEGG databases. Since bacteria and T cells compete for nutrients and energy (Kedia‐Mehta and Finlay 2019), the metabolic changes caused by the altered composition of the gut microbiota in Cpd1‐treated EAE rats may have influenced the function of pathogenic autoreactive T cells in the gut‐associated lymphoid tissue, thereby ameliorating the disease.

Given data showing that bacterial peptidoglycan can serve as a cofactor for the development of CNS autoimmune diseases (Visser et al. 2005), our finding of an enhanced pathway for the synthesis of peptidoglycan (K07259) in the Cpd1‐treated group would predict a more efficient function of antigen‐presenting cells and an enhanced Th1 response. However, this was not the case, as the frequency of Th1 cells in the lamina propria was similar in Cpd1‐treated and untreated EAE rats (Lazarević et al. 2024). It is noteworthy that a reduced lipopolysaccharide biosynthesis pathway (K02840) in Cpd1‐treated EAE rats could contribute to a lower stimulation of innate immune cells and, thus, to a lower severity of EAE.

In our previous study, the genus *Turicibacter*, which also belongs to the *Erysipelotrichaceae* family and the phylum *Firmicutes*, was recognized as a potential biomarker of the gut microbiota for resistance to EAE induction in rats (Stanisavljević, Lukić, Momčilović, et al. 2016). It was only found in the EAE‐resistant AO rat strain but not in the EAE‐susceptible DA rat strain. In the present study, *Turicibacter* was also undetectable in EAE rats regardless of treatment with Cpd1. The species *Ileibacterium_valens*, which was enriched in the microbiota of Cpd1‐treated EAE rats, also belongs to the *Erysipelotrichaceae* family but has not yet been investigated in connection with autoimmune diseases. However, a more recent study showed that the genus *Ileibacterium* was enriched in oxymatrine‐treated EAE mice and correlated negatively with neurological score, suggesting a positive effect on the disease (M. L. Zhang et al. 2023). The beneficial effect of antibiotic treatment in rats with experimental autoimmune neuritis was accompanied by an increased abundance of the family *Sutterellaceae* and the genus *Parasutterella* (Sprenger‐Svačina et al. 2024), which we also found in Cpd1‐treated EAE rats. Surprisingly, the only species that was decreased in Cpd1‐treated EAE rats was *Lactobacillus murinus*, which belongs to a probiotic strain previously associated with resistance to EAE induction (Stanisavljević, Lukić, Soković, et al. 2016). Nevertheless, the balance between the different bacterial taxa is most likely crucial for the modulation of EAE by a treatment that directly or indirectly affects the gut microbiota.

In conclusion, our study shows that amelioration of EAE produced through stimulation of FFAR2 signaling early on in the course of disease is associated with changes in the composition of intestinal microbiota. There are several limitations of our study. First, gut microbiota was sampled from the terminal ileum only, and it will be important to determine its effects on the composition in other relevant parts of the intestinal tract. Nonetheless, our prioritization of the terminal ileum stems from the fact that this is a key site for the interaction between gut microorganisms and local immune cells and, in particular, for the generation of Th17 cells known to have consequent effects on CNS autoimmunity (Nouri et al. 2014; Ivanov et al. 2009). A second study limitation is that SCFAs were examined in the cecum only, and therefore, these results should be corroborated through extended analysis of SCFA concentrations in other parts of the intestine. The focus on the cecum in this initial study was based on it being a source of ample material for SCFA determination and its proximity to the terminal ileum. A further potential limitation is that samples were obtained at only one‐time point, and therefore, it remains interesting to determine the effects of treatment on the composition of gut microbiota and SCFA at later time points. However, in this study, we wanted to have the samples at the same time as the intestinal samples obtained in our previous study for analysis of small intestine immune cells in order to obtain comparable data. Despite these limitations, our study provides solid data indicating that changes in gut microbiota achieved through FFAR2 agonism in the intestine in the inductive phase of EAE contribute to the previously observed amelioration of the disease by the treatment. Thus, it is plausible to conclude that the beneficial effects of Cpd1 in CNS autoimmune disease are multifactorial.

## Author Contributions

Bojan Jevtić: writing‐original draft, methodology, investigation, data analysis. Goran Stegnjaić: methodology, investigation, writing‐review and editing. Suzana Stanisavljević: methodology, investigation, writing‐review and editing. Milica Lazarević: methodology, investigation, writing‐review and editing. Filip Nikolić: methodology, writing‐review and editing. Graeme L. Fraser: conceptualization, writing‐review and editing. Đorđe Miljković: conceptualization, writing‐original draft, funding acquisition, supervision, project administration. Mirjana Dimitrijević: conceptualization, writing‐original draft, supervision, data analysis.

## Ethics Statement

The experiments were performed with permission granted by the Veterinary Administration of the Ministry of Agriculture, Forestry and Water Management of the Republic of Serbia (Nos. 323–07‐01337/2020–05 and 323–07‐05815/2020–05/1).

## Conflicts of Interest

The authors declare no conflicts of interest.

### Peer Review

The peer review history for this article is available at https://publons.com/publon/10.1002/brb3.70350.

## Supporting information



Supporting data

## Data Availability

The data that support the findings of this study are available from the corresponding author, Đ.M., upon reasonable request.
